# Expanding the Availability of Scalp Cooling to All Patients at Risk of Chemotherapy-Induced Alopecia

**DOI:** 10.3390/jmahp12030013

**Published:** 2024-07-10

**Authors:** Portia Lagmay-Fuentes, Andrea Smith, Shannon Krus, Laurie Lewis, Asma Latif, Tiffany Gagliardo, Manpreet Kohli

**Affiliations:** 1 RWJ Barnabas Health, Hamilton Township, NJ 08690, USA; 2 Memorial Sloan Kettering Cancer Center, New York, NY 10065, USA; smitha2@mskcc.org; 3 Washington University Siteman Cancer Center, St. Louis, MO 63110, USAlaurielewis@wustl.edu (L.L.); 4 Oncologist, Sturdy Memorial Hospital, Attelboro, MA 02703, USA

**Keywords:** alopecia, insurance-based billing model, reimbursement, scalp cooling, self-pay

## Abstract

Alopecia is an undesirable side effect of cancer chemotherapy. The mitigation of alopecia is a desirable adjunct treatment for patients with cancer. FDA-cleared scalp cooling (SC) devices have been successfully used to prevent or reduce chemotherapy-induced alopecia (CIA). This paper provides an understanding of the implementation and value of the new Insurance-Based Billing Model used in the USA for SC and its benefits compared with the original self-pay model. This improved compensation change will result in all patients in need, including underserved and disadvantaged populations, receiving equitable healthcare by allowing access to this valuable supportive care technology.

## 1. Introduction

### 1.1. Background

Chemotherapy-induced alopecia (CIA) is a frequent side effect of systemic chemotherapy [1]. CIA is distressing for patients, as shown by results of various quality of life (QoL) assessments [1]. Hair loss affects self-esteem, is a continuous reminder of the disease, affects the patient’s privacy, and negatively impacts social and work interactions [2]. The impact of CIA on patients ranks highest, ahead of fatigue, nausea, trouble sleeping, and early hot flashes [3]. Among the procedures to reduce or mitigate CIA (e.g., vitamin D3 applications and immunomodulatory tellurium), only scalp cooling (SC) has demonstrated efficacy [4]. In 2015, the FDA approved the DigniCap as the first device for SC; the Paxman scalp cooling system was approved in 2017 [5,6]. Both are indicated to reduce the likelihood of CIA in patients with solid tumors such as breast, ovarian, colorectal, bowel, and prostate cancer [6] tumors and have been widely used to prevent or reverse hair loss [1,7,8].

Evidence for the efficacy of SC in mitigating CIA comes from several sources [8,9]. Results from a systematic review and meta-analysis that included nearly 1100 participants (mostly patients with breast cancer) found that SC significantly reduced the risk of CIA [9]. The National Comprehensive Cancer Network (NCCN) includes SC in its guidelines for treating breast and ovarian cancers [10,11,12] as a category 2A recommendation to reduce or prevent CIA. For patients with breast cancer, NCCN recommends SC to reduce the incidence of CIA for patients receiving chemotherapy, including in neoadjuvant and adjuvant settings [10]. For patients with ovarian cancer, NCCN recommends SC for patients on regimens associated with high rates of CIA [11].

### 1.2. Mechanism of Action

**Figure 1:**
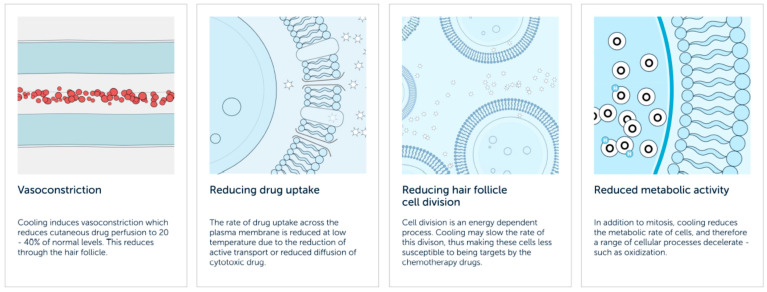
Proposed mechanisms for scalp cooling. Legend: Scalp cooling affects hair follicles (keratinocytes) in ways that lessen the uptake and activity of chemotherapeutic agents, thereby reducing hair loss [13].

There is evidence that SC reduces the uptake of chemotherapeutic agents by human keratinocytes [13] due to the vasoconstriction of vessels (Figure 1), although the exact mechanism according to which SC prevents or reduces CIA is unknown. Additionally, it is thought that vasoconstriction from cooling reduces the metabolic rate of keratinocytes, which also reduces drug uptake and hair follicle cell division (Figure 1) [14,15].

### 1.3. Clinical Data in Support of SC

SC has been shown to prevent or reduce CIA in numerous US studies and studies from the rest of the world [16,17,18,19,20,21] and is considered the most effective method for treating CIA [22]. Table 1 summarizes clinical trials using the Paxman and Dignitana scalp cooling systems.

In 2017, the FDA cleared the expanded use of the Dignitana DigniCap Cooling System for patients with solid tumors [5]. In 2018, the FDA cleared the use of the Paxman SC system for breast cancer and all other solid tumors [6,7]. Furthermore, the AMA resolved to seek broad insurance coverage for SC to improve the lives of patients with cancer [28].

### 1.4. Use of the SC System

**Figure 2:**
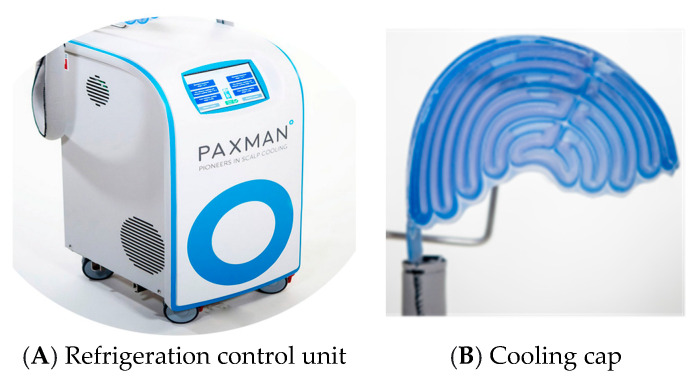
The Paxman scalp cooling system. Legend: The Paxman scalp cooling system consists of a refrigeration unit (**A**) and a cap to fit on the scalp (**B**) [31].

The system consists of a compact refrigeration unit containing coolant that is circulated through the cooling cap (Figure 2) [29]. The coolant passes through channels in the cap (with six sizes available for the best fit). Its instant cooling capability once connected to the system allows for its immediate use by the patient. Temperature sensors ensure the cap maintains an even and consistent temperature on the scalp. A neoprene cover insulates the head from high room temperatures while absorbing condensation. Bungee-cord adjustments ensure a close fit to the patient’s scalp [30]. The Dignitana DigniCap functions in a similar manner but uses a single-size adjustable cap [30].

Patients use the system by appointment at their cancer treatment sites [31]. Introducing SC to eligible patients requires an integrated effort on the part of physician, nursing, and care manager medical teams to educate patients on the benefits and risks of SC, set appropriate expectations for the outcomes, and monitor and manage the effect of SC intervention [32]. Protocols for its use vary by hair type and are detailed in the patient guide for use [33]. The Paxman scalp cooling system is operated by a member of the patient’s healthcare team; patients are fitted for a cap and then bring the cap to all their chemotherapy sessions. Patients wear the cap for 30 min preinfusion, throughout the alopecia-causing chemotherapy session, and for a period of 90–120 min afterwards [34].

### 1.5. The Impact of CIA on Patients

Among the side effects of chemotherapy, CIA is one of the most distressing and most feared [35,36]. Up to 8% of female patients with cancer have refused treatment because of the expected CIA [37]. For patients with breast cancer, the physical, emotional, and psycho-social impacts of CIA play negative roles in their QoL and ability to cope with their disease and its treatment [1,36]. Often, patients find wigs or scarves uncomfortable, especially if they experience hot flashes due to treatment [4]. In addition, wearing wigs or scarves can lead to a loss of anonymity, as these distinguish those with hair loss (i.e., likely patients with cancer) from those without, thereby affecting their interactions with others [38,39]. SC has been shown to improve QoL for most patients with cancer [4,15].

Although SC is considered the most effective method available for mitigating CIA, barriers to its full clinical utility remain. For example, one study showed that whereas 62% of providers favored recommending SC to their patients, only 26% initiated a discussion with patients regarding SC [40]. Financial concerns were most often cited as a reason for not discussing SC [41].

## 2. The Current Utilization of Scalp Cooling

Over 1000 locations across 46 states in the US, including 43 NCCN and NCI-designated comprehensive centers, offer SC by Paxman and Dignitana [12]. Sales data from both Dignitana and Paxman reflect 19,893 patients received 127,437 scalp cooling treatments between 1 January 2021, and 30 September 2023. Over 11,000 patients in the US have used SC since 2017, and the number of patients using SC is growing [28]. Until June 2022, SC was only available as a self-pay service [28].

Although the efficacy of SC in reducing or preventing CIA has been established, its cost has reduced its availability, especially to underserved patient populations. SC costs approximately USD 1200 to USD 1800 per chemotherapy course, and insurance coverage is not routine. Patients are therefore more likely to use SC if they have private insurance or live in zip codes with average incomes of USD 100,000. Nonprofit organizations have emerged to combat this care inequity by helping to fund SC [40].

One study showed that financial concerns were cited by 58% of responders as the primary reason for not recommending SC to patients [40]. A study of patients with breast cancer found significant financial debt from out-of-pocket expenses resulting in decreased QoL [41]. Some organizations, such as HairToStay, have been established to help patients lessen the financial burden of paying for SC [42,43]. Reducing the financial barrier to SC for all patients with cancer from all socioeconomic levels is needed.

Coverage by medical insurance is a way patients can reduce their financial barriers. The largest payor in the US is Medicare; in the fiscal year of 2021, Medicare processed over 1 billion fee-for-service claims [44]. According to the LCD, “the use of a scalp hypothermia device that has been approved by the FDA for the prevention of CIA shall be considered reasonable and necessary for patients with solid tumors” [45].

## 3. Billing and Coding Guide

In 2021, Paxman began a process to help open access to Paxman scalp cooling systems to any U.S. patient, regardless of insurance coverage or financial situation. Under this new system, any patient receiving solid tumor chemotherapy that is likely to cause alopecia will have a better chance of receiving SC coverage either through their insurance or another method. In 2023, 82% of patients received positive coverage, and 86% of patients without coverage using Paxman’s Hub services under the new Insurance-Based Billing Model were supported by the patient assistance program [46]. The Insurance-Based Billing Model is further explained in Section 3.3.

### 3.1. ICD-10-CM Diagnosis Codes

The Paxman scalp cooling system is approved for use on patients receiving chemotherapy that is likely to cause alopecia, especially taxanes and anthracyclines. Trials were carried out on patients who had solid tumors, such as ovarian, breast, colorectal, and prostate cancer. All diagnosis codes are expected to correspond to the patient’s condition at the full discretion of the physician. The ICD-10 codes C50 for breast cancer and C56 for ovarian cancer are used. There is no separate ICD-10 code for scalp cooling.

### 3.2. CPT Codes for SC

As of November 2021, the CPT codes for scalp cooling are 0662T and 0663T.

- 0662T: Scalp cooling mechanical; initial measurement and calibration of cap. This code is billed when the cap is fitted to the patient and may only be used one time per patient.
- 0663T: Placement of the device, monitoring, and removal of the device. This code is billed every time the patient receives SC during chemotherapy and is to be used in conjunction with chemotherapy administration codes 96409, 96411, 96413, 96415, 96416, and 96417.

These two new codes are used by providers when billing insurance companies. In February 2023, Dignitana and Paxman formally requested the development of a local coverage determination (LCD) within Palmetto for SC for the treatment of CIA and requested that Palmetto establish a physician fee schedule for CPT codes 0662T and 0663T. Paxman currently serves 55 locations and Dignitana serves 37 locations within the Palmetto GBA jurisdiction [47,48]. On 3 October 2023, the Palmetto GBA Medicare Administrative Contractor (MAC) deemed SC a reasonable and necessary service and issued the first LCD (covering seven states in the southern US: AL, GA, NC, SC, TN, VA, and WV), thus providing a pathway for the reimbursement of Medicare scalp cooling claims for patients in the seven-state service area. There are other insurance companies in the region. Some private insurance companies pay claims in some areas, but it differs by area and region. Private insurers often look to Medicare to help inform their decisions, and some pay based on the assigned CPT codes and APC rate—increased communication and advocacy with private insurers would also likely increase the chances of private insurers paying. The plan is to pursue the additional MACs to advocate their implementation of an SC LCD now that there is another active LCD paving the way that can be used as a model. The next step is an advocacy strategy for working with the other remaining MACs—likely one or two at a time over the next few years to implement positive LCDs for SC and continue to engage with private payers to advocate covering scalp cooling.

### 3.3. Definition of the Insurance-Based Billing Model

The Insurance-Based Billing Model is a process of acquiring specialty care or treatment that providers administer on site in their practices. Rather than relying on a specialty pharmacy or another vendor, the healthcare provider purchases, stores, and then administers the product or service to a patient [49]. Under patient self-pay, the patient purchases caps directly from the manufacturer. Through the patient self-pay model, the cost to the patient is based on the number of treatments the patient receives. Treatment 1 (including the personal cap kit) is USD 350, treatments 2 through 4 are USD 350 each, treatments 5 through 6 are USD 200 each, and treatments 7 through 12 are USD 100. Pricing is capped at USD 2400—patients will pay no more than this, no matter how many cycles they receive [30].

In terms of reducing the out-of-pocket costs to patients, Paxman is the leader in driving affordable SC and eliminating disparities in the availability of this supportive care. As of 2022, the reimbursement model for Paxman SC was changed to an Insurance-Based Billing Model [29].

## 4. Advantages of the Insurance-Based Billing Model vs. Self-Pay for Providers and Patients

The Insurance-Based Billing Model reimbursement model for SC provides several benefits, including allowing healthcare providers to control all aspects of administration of the service [49], the ability to provide the procedure at any visit, including the first, and increased revenue for providers [50]. Moreover, the Insurance-Based Billing Model makes it easier for providers to submit claims to insurers, and it allows for more positive payor coverage from commercial insurance and federal programs, including Medicare or Medicaid [50,51,52].

Furthermore, the Insurance-Based Billing Model addresses financial uncertainty and makes SC a type of supportive care available to all patients, independent of socio-economic status. A robust patient assistance program (PAP) ensures that uninsured or underinsured patients are also able to receive SC with their chemotherapy treatment [29]. As such, the Insurance-Based Billing Model enables three pathways to access: insurance coverage, enhanced patient assistance at 600% of the federal poverty level, or direct billing of the patient by the providing site.

**Figure 3:**
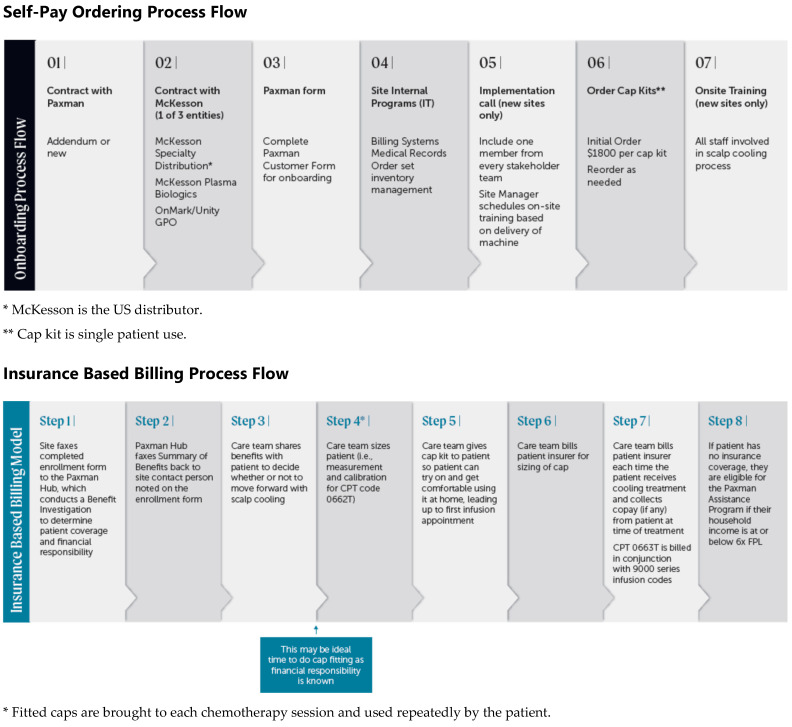
The self-pay model and the new onboarding and insurance-based billing model.

The step-by-step process using the old self-pay model and the Insurance-Based Billing Model for SC and Hub services are depicted in Figure 3. The Hub serves as the patient and reimbursement support service center. It is a call center offering patient advice and support, payment and Rx processing under the self-pay model, and reimbursement support to providers under the Insurance-Based Billing Model.

The disadvantages of the Insurance-Based Billing Model include the financial risk to providers. Cap kits have an associated upfront cost, which is not a guaranteed return [50]. Additionally, providers must manage inventory, such as various sizes of SC caps [50]. However, it is clear the advantages greatly outweigh the disadvantages of the Insurance-Based Billing Model.

As such, the new Insurance-Based Billing Model continues to be implemented to help open access to Paxman SC to any US patient regardless of their insurance coverage or financial situation. As noted above, PAP support is set at six times the federal poverty level based on household income and size. Patients and providers can access SC through the hub service which is offered through CoverMyMeds, a McKesson company [46] that undertakes the full benefits investigation. The Paxman Hub is a call center with specially trained case managers who work with patients to provide information on payer coverage, coding, and the PAP. The Hub case managers are also there to assist HCPs, physicians, nurses, and all healthcare professionals with any questions they may have. This service is unique to Paxman and is not offered by Dignitana.

## 5. Switching from Self-Pay to the Insurance-Based Billing Model in Centers Already Using Scalp Cooling with a Self-Pay System

For centers that have offered SC with the self-pay reimbursement model, the switch to the Insurance-Based Billing Model can take 6–9 months and involves multiple stakeholders, including clinical staff (RNs and MDs), administration, compliance, regulatory affairs, billing, patient financial services, the supply chain director, and legal representatives. The identified project team has bi-monthly or weekly meetings to (a) understand the different clinical and financial roles and responsibilities, (b) establish the documentation needed to bill insurance or the Centers for Medicare & Medicaid Services (CMS), (c) determine the nursing notes needed to back the claim, (d) ensure the enrollment form is appropriate, (e) perform a cost analysis to determine how much to bill, (f) determine how to operationalize everything to make it as seamless as possible across the entire organization, (g) establish the workflow, coordinating the efforts of all departments, (h) perform a benefits review, (i) update policies and procedures, (j) determine how to manage supplies, (k) update the templates for electronic medical record (EMR) orders, (l) coordinate the education of clinical staff, and (m) update patient educational material.

As with any major policy or protocol change, there are challenges and obstacles that must be overcome. For the Insurance-Based Billing Model switch, some of the important challenges included determining which insurance companies cover SC and which do not, establishing internal protocols, coordination to manage chair and staff time, working out the ordering process, dealing with international patients who are on self-pay, working with patients already in treatment who are following the self-pay model, managing the increased demand for SC, and educating patients regarding the new financial structure.

For centers just starting SC with the Insurance-Based Billing Model without ever having used self-pay, the internal team might involve senior leaders, clinical staff, the oncology unit director, and team members from the supply/ordering department, from the EMR section to build order sets, and from insurance billing departments. The coordination of the workflow happens simultaneously with the signing of a contract, ordering and receipt of SC machines, and education of staff by a company representative.

## 6. Summary and Importance of the Insurance-Based Billing Model

Although self-pay is more familiar and thus more convenient for the staff at centers offering SC, the centers that have implemented the Insurance-Based Billing Model believe that regardless of how difficult it may seem, it is a very important endeavor and results in positive changes for patients and for staff. They do not have to worry about how supportive care is paid for. Insurance-Based Billing gives patients some control over CIA, which they could not control in the past. Most importantly, it enables access for patients in underserved communities. Lastly, it delivers a message to payers that managing CIA is very important for patients—that this is not just a cosmetic issue but rather impacts the psychological and physical well-being of patients.

Reports from sites using the Insurance-Based Billing Model for SC indicate that more patients are utilizing SC because of the reduced financial burden. Access to SC has increased now that patients are not paying out of pocket [46].

## Figures and Tables

**Table 1:** Clinical trials using scalp cooling.

Title/NCT#1/Status	Cancer Type; (N); Objective	Results	Reference
Dutch observational followed by randomized study/completed No NCT Number	Breast cancer; (N = 53); potential optimum post-infusion cooling times (PICTs) in patients receiving docetaxel	A total of 81% of scalp-cooled patients did not require head covering vs. 27% of non-scalp-cooled patients. A 45 min PICT can be recommended in 3-weekly docetaxel regimens.	[16]
Pivotal trial for Dignitana pivotal study NCT01831024/completed	Stage I/II breast cancer; (N = 117); efficacy and safety and patient satisfaction with DigniCap SC	Hair loss prevented by DigniCap in 66.3% of patients with breast cancer receiving adjuvant chemotherapy, vs. a control group where all patients experienced significant hair loss.	[15]
Retrospective DigniCap single-arm study	204 patients with Stage I-V breast (n = 120), ovary, lung, uterus, esophagus, prostate, chest, urethra, rectum, larynx, bladder, colon, or liver cancer and non-Hodgkin’s lymphoma	A total of 84% had <50% hair loss with DigniCap. At follow-up, there were no side effects or scalp metastasis present.	[23]
Prospective, non-randomized with DigniCap	Women with breast, endometrial, or ovarian cancer (n = 55) Breast cancer: 35 adjuvant (63.6%) 5 palliative (9.1%) 2 neo-adjuvant (3.6%) Ovarian cancer: 12 (21.8%) Endometrial cancer 1 (1.8%)	56% with <50% hair loss 1.8% could tolerate SC	[24]
SCALP Study/ NCT01986140/completed	Stage I/II Breast cancer; (N = 236); to determine that Paxman SC is safe and effective in reducing CIA	Successful hair preservation was found in 50.5% of women with cooling compared with 0% of women in the control group.	[25]
HOPE Study/no NCT# (Japanese)/completed	Breast cancer; (N = 48); to evaluate the proportion of patients with no alopecia at the end of chemotherapy	More patients had no alopecia at the end of chemotherapy in the scalp-cooling group than in the control group (26.7% vs. 0%; *p* = 0.011). 85.7% of patients in scalp cooling group and 50.0% in the control group with alopecia had a hair volume increase ≥50% within 12 weeks after chemotherapy.	[26]
RCT of scalp cooling for prevention of CIA/CTRI/2017/02/007896/completed ^1^	Breast cancer; (N = 51); to assess the effect of scalp cooling on CIA	Hair preservation was higher in SC vs. control arm (56.3% vs. 0%, *p* = 0.000004). Hair regrowth was higher in SC arm vs. control at 6 weeks (89% vs. 12%; *p* < 0.001) and 12 weeks (100% vs. 59%, *p* = 0.0003). Loss of hair at the primary endpoint was lower in SC vs. control arm (45% vs. 82%, *p* = 0.016).	[20]
Efficacy of Paxman scalp cooling in preventing CIA in Black patients with breast or gynecological cancers/NCT04626895/completed	Breast and gynecological; (N = 15); to measure the efficacy of scalp cooling with the Paxman scalp cooling device in a diverse patient population	Data will be reported by no later than June 2025 for patients treated with Sacituzumab govitecan, eribulin, and Trastuzumab deruxtecan.	[27]
Prospective observational scalp cooling to prevent CIA in patients with breast cancer/ no NCT#/completed	Breast cancer; (N = 131); to assess whether a scalp cooling device is effective in reducing CIA and assess adverse treatment effects	Hair preservation was successful in 71.0% of women who underwent scalp cooling for anthracycline/taxane-based chemotherapy or taxane monotherapy.	[19]
Open-label, prospective, nonrandomized trial in patients with breast cancer/NCT01008774	Breast cancer; (N = 238); assess two different methods of scalp cooling to reduce CIA in docetaxel-treated patients	Alopecia occurred with PAX, a cold cap (CC), and no cooling under 3-weekly docetaxel in 23%, 27%, and 74% and under weekly docetaxel in 7%, 8%, and 17%, respectively. Overall, cooling (PAX and CC combined) reduced the risk of alopecia by 78% (HR 0.22; 95% CI 0.12 to 0.41).	[17]

^1^ The RCT published by Bajpai et al. [20] has a clinical registration # instead of an NCT #.

## Data Availability

Data sharing is not applicable to this article as no datasets were generated or analyzed during the current study.

## Abbreviations

APC	Ambulatory Payment Classification
CIA	chemotherapy-induced alopecia
CMS	Centers for Medicare & Medicaid Services
EMR	electronic medical record
FDA	Food & Drug Administration
LCD	local coverage determination
MAC	Medicare Administrative Contractor
MBC	metastatic breast cancer
NCCN	National Comprehensive Cancer Network
NCI	National Cancer Institute
QoL	quality of life
PAP	patient assistance program
RCT	randomized clinical trial
SC	scalp cooling
